# Microscopic Mechanism of Glass Surface Activation, Annealing and Etching on Glass–Ti/Cu Interfacial Adhesion

**DOI:** 10.3390/mi17070836

**Published:** 2026-07-14

**Authors:** Tailong Shi, Wending Yang, Qi Li, Jingxuan Yang, Zhonghao Li, Hua Hong, Zhong Zhang, Guodong Zhang, Andrew C. Chang

**Affiliations:** 1 Department of Integrated Circuits, Southeast University, Nanjing 211189, China; yangwending@seu.edu.cn (W.Y.); 220256680@seu.edu.cn (Q.L.); 230259689@seu.edu.cn (J.Y.); 220256612@seu.edu.cn (Z.L.); huahong@seu.edu.cn (H.H.); 2 Jiangsu Silicon Integrity Semiconductor Technology Corporation (JSSI), Nanjing 211800, China; simon.zhang@jssisemi.com (Z.Z.); jerry.zhang@jssisemi.com (G.Z.); 3 AaltoSemi, Nanjing 211806, China; andrew.chang@aaltosemi.com

**Keywords:** glass substrate, adhesion strength, annealing, advanced packaging

## Abstract

Due to its excellent electrical and thermal performance, glass packaging demonstrates significant potential in heterogeneous integration of chiplets advanced packaging system, but limited by its poor interfacial adhesion strength between glass and metals. This article studies the mechanisms of glass–metal bonding interface at the microscale level, and the adhesion strength at the macroscale level. In detail, the changes of the adhesion strength after glass surface activation, annealing and micro-etching processes were characterized, and the correlation between the microscale mechanisms and the macroscale adhesion variations of each process was studied. X-ray photoelectron spectroscopy (XPS) results indicate that the increase in Si-OH bond is the key to glass surface activation. Fourier transform infrared spectroscopy (FTIR) was applied to quantitatively correlate the dynamic evolution of surface polar hydroxyl groups on glass substrates with the subsequent glass–metal interfacial bonding strength, and verified the conclusion above. The adhesion strength increased by 2.3 times after surface activation, and by 4.1 times after annealing, while it decreased slightly after etching. Furthermore, the glass–Ti seed layer interface was studied at the atomic level to better analyze the changes in macroscopic adhesion. XPS depth profiling confirmed the formation of Si-O-Ti bonds at the glass–Ti interface, which may contribute to the enhanced adhesion. After annealing, X-ray diffractometer (XRD) characterization revealed the great change in grain structure caused a reduction in residual stress within the plated layer.

## 1. Introduction

With Moore’s law approaching its physical limits, advanced packaging technology has become a critical pathway for continuously enhancing the performance of electronic systems [1,2,3,4]. In recent years, glass packaging has become an ideal candidate for next-generation high-density packaging, owing to their superior electrical properties, potential for ultra-high interconnect density, and cost-effectiveness [5,6,7,8,9,10,11,12]. However, glass is inherently a material with stable chemical properties. Its smooth surface makes it difficult to form robust bonding with metal atoms, resulting in weak adhesion between glass and metal [13,14,15]. This can lead to delamination or peeling between the metal circuitry and the glass substrate, causing electrical connection failures [16,17,18,19].

To address these bottlenecks, academic research has primarily focused on two approaches to improve interfacial adhesion strength. One approach involves introducing an interlayer between the glass and the metal to enhance adhesion. For instance, Kuwahara et al. used chemical bath deposition to prepare a Cu(OH)_2_/Cu(O,S) bilayer film on glass [13]. This bilayer was then chemically reduced to a metallic copper layer using an aqueous NaBH_4_ solution. The resulting Cu/Cu(OH)_2_/Cu(O,S) tri-layer structure exhibited an average adhesion strength of 4.4 N/cm. However, the copper layer detached completely once the bilayer film was fully reduced. Yang et al. synthesized 3-Aminopropyl triethoxysilane-capped Pd nanoclusters (APTES-Pd) and combined it with a porous titanium dioxide (TiO_2_) adhesion-promoting layer, achieving an adhesion strength of 1.96 gf/cm for the electroplated copper layer [15]. Xu et al. proposed a method using a sol–gel dip-coating process to prepare a silver-doped zinc oxide (SZO) thin film on a glass substrate as an adhesion layer [20]. Using zinc acetate and silver acetate as precursors, they prepared a silver-doped zinc oxide sol. Through dip-coating, pre-sintering, and high-temperature annealing, an SZO film was formed on the glass surface, resulting in an adhesion strength of 3.21 N/cm for the electroplated copper layer. Similarly, Ghosh et al. improved the adhesion of the copper layer to the metal by preparing a zinc oxide adhesion layer on glass via a wet chemical method [14]. However, when it comes to the preparation of adhesion layers, the fabrication process can be relatively complex and often involves high-temperature sintering to achieve proper bonding of the adhesion layer, which may pose challenges for integration into existing packaging production lines [21,22,23]. In addition, controlling the thickness and uniformity of solution-processed adhesion layers can be difficult.

The other approach involves pretreating the glass to modify its surface physical morphology or chemical state, thereby enhancing the bonding between glass and metal to improve adhesion. Pandey et al. used ultrasonic machining (USM) or electrochemical discharge machining (ECDM) to locally roughen glass substrates, creating numerous micropits and groove structures on the surface [24]. The maximum surface roughness reached 2.48 µm (Ra), which facilitated the embedding of the electroplated copper layer and the formation of mechanical interlocking. In cross-hatch adhesion tests, the adhesion strength of a 16 µm thick copper layer improved to 5B. Huang et al. increased surface roughness (0.14 µm) through mechanical abrasion with sandpaper followed by etching with hydrofluoric acid and piranha solutions [25]. The subsequently prepared silver seed layer via silver ammonia solution reduction exhibited an adhesion strength of 5B. Yang et al. treated fused silica glass substrates with air-based cold plasma, investigating the effect of plasma treatment duration on surface properties and the adhesion performance of copper films [26]. In tape peel tests, after 25 s of plasma pretreatment, the residual proportion of the copper layer increased from approximately 80% to 98%. Bakar et al. found that immersing glass in concentrated sulfuric acid, thoroughly rinsing with deionized water, and ultrasonically cleaning with ethanol most effectively removed organic contaminants and improved the hydrophilicity of the glass [18]. The silver film deposited via physical vapor deposition (PVD) showed no peeling after ultrasonic stripping tests. However, in the context of advanced packaging applications, surface roughness at the submicron or micron level can be less favorable for achieving uniform seed layer deposition inside vias and may also affect the morphology of metal traces in the RDL [27,28,29,30]. Furthermore, physical damage caused by surface roughening processes may lead to microcracks, which could potentially contribute to glass fracture [31,32].

Both approaches provide possible solutions to increase the adhesion strength between glass and metal. However, detailed atomic-scale characterization of the activated glass surface and its bonding mechanisms remains limited. The change of the chemical state at the glass–metal interface after metal deposition has received little attention, and the effects of annealing and etching on interfacial adhesion have not been extensively discussed.

To address these aspects, this study employs atomic-scale characterization to investigate the effects of oxygen plasma treatment on the chemical state and elemental composition of glass surfaces, and systematically elucidates the underlying modification mechanism. The evolution of the chemical state at the glass–metal interface after seed layer sputtering is further examined, and the influence of post-process steps (seed layer etching and annealing) on interfacial adhesion is evaluated. Key findings show that plasma treatment induces surface hydroxylation, increasing the relative Si-OH content up to 36%. FTIR analysis, applied on the surface of glass substrate, confirms the change of Si-OH content, consist with the XPS results. The transformation from Si-OH groups into Si-O-Ti covalent bonds after Ti deposition, correlating with the enhanced macroscopic adhesion. Moreover, seed layer etching reduces adhesion, whereas annealing increases it, with the maximum adhesion strength reaching 4.1 times the initial value. These results provide mechanistic insights into glass–metal interfacial modification, and can offer reference for related research in advanced packaging.

## 2. Materials and Methods

### 2.1. Glass Surface Pretreatment

Schott BF33 glass substrates (50 mm × 50 mm × 0.5 mm) were first cleaned to remove surface contaminants. This was done by sequential ultrasonic cleaning in isopropanol, anhydrous ethanol, and deionized water, each for 15 min. Immediately afterwards, the substrates were dried using a nitrogen gun. Surface treatment was then performed using a vacuum plasma cleaner (SINO-Plasma 100, Yiwentech Co., Ltd., Wuxi, China) with oxygen plasma. The plasma treatment parameters were set as follows: power of 300 W, gas flow rate of 300 sccm, chamber pressure of 0.5 Torr, and temperature of 150 °C. Treatment durations of 0.5 min, 1 min, 2 min, and 4 min were investigated.

### 2.2. Glass Surface Metallization

Following plasma treatment, a seed layer consisting of 200 nm Ti and 400 nm Cu was deposited onto the glass surface using a reciprocating magnetron sputtering system (CS-200z, MAKO SEMI, Chigasaki, Kanagawa, Japan). Direct current electroplating was subsequently carried out using a high-precision digital source meter (IT2806, ITECH, Shenzhen, China). The plating solution consisted of 250 g/L copper sulfate and 20 g/L concentrated sulfuric acid. Plating was performed at a current density of 4 A/dm^2^ (amperes per square decimeter, ASD) with mechanical agitation at 1000 rpm. Copper strips with a thickness of 30 μm were electroplated. Three such strips, each measuring 10 mm × 50 mm with a 5 mm gap between them, were patterned on each substrate.

### 2.3. Seed Layer Etching and Annealing

**Figure 1:**
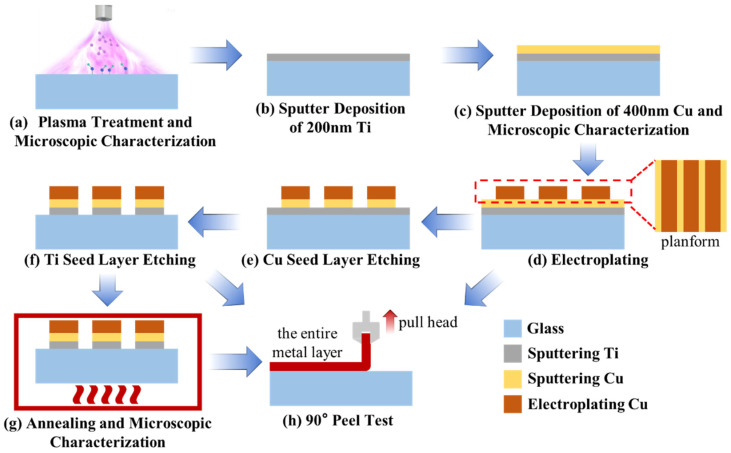
Process Flow Diagram: (**a**) Plasma Treatment; (**b**) Ti Seed Layer Deposition; (**c**) Cu Seed Layer Deposition; (**d**) Electroplating; (**e**) Cu Seed Layer Etching; (**f**) Ti Seed Layer Etching; (**g**) Annealing; (**h**) Macroscopic Adhesion Strength Test: 90° Peel Test.

Post-electroplating, samples underwent seed layer etching and annealing. Wet chemical etching was used for seed layer removal. Samples were sequentially immersed in copper etchant and titanium etchant (Nitric acid mixture, RunMa Electronic Material, Wuxi, China) for 5 min and 1 min, respectively. After each etching step, samples were rinsed thoroughly with deionized water and dried. Annealing was performed in a tube furnace (KJ-T1200-S6030LK, henanhonglu, Zhengzhou, China). Samples were placed in the furnace, which was then evacuated and backfilled with argon to 70 kPa as a protective atmosphere. The furnace temperature was ramped from room temperature to 200 °C at a rate of 5 °C/min, held for one hour, and then allowed to cool naturally to room temperature. The complete process flow is illustrated in Figure 1.

### 2.4. Characterization

The water contact angle of the glass was measured and analyzed using a contact angle goniometer (OCA25, Dataphysics, Filderstadt, Germany) and ImageJ (v1.53t) software. The chemical states of surface elements on the glass were acquired via X-ray photoelectron spectroscopy (XPS; ESCALAB QXi, Thermo Fisher Scientific, Waltham, MA, USA). All measurements were performed using a monochromatic Al Kα X-ray source with a photon energy of 1486.6 eV, operated at an anode voltage of 15 kV and an output power of 150 W under an ultra-high vacuum of 5 × 10^−10^ Torr. The binding energies were calibrated for surface charging effects by referencing the adventitious carbon C 1s peak at 284.8 eV. All core-level spectra were fitted with Shirley-type and linear backgrounds for quantitative deconvolution analysis. Surface morphology and roughness of the glass were characterized using an atomic force microscope (AFM; ICON, Bruker, Billerica, MA, USA). All measurements were performed in tapping mode with a scan size of 500 nm × 500 nm. For each sample, 5 independent regions on the glass surface were randomly selected for scanning to eliminate test deviations caused by surface inhomogeneity. The average values and corresponding standard deviations of Ra and Rq were calculated from all repeated measurements. FTIR spectra of the glass surface were acquired using a Vertex 80v spectrometer equipped with a HYPII infrared microscope (Bruker, Billerica, MA, USA). The adhesion strength between the copper strips and the glass was quantitatively evaluated using a 90° peel test performed on a high-precision tensile/compression testing system (Dage 3800plus, Nordson, Westlake, OH, USA). One end of a copper strip was initially delaminated with a surgical blade. This pre-peeled end was then gripped by the system’s pull head, which subsequently retracted at a constant peel rate of 100 µm/s. The sample stage moved synchronously at the same speed to maintain a constant 90° angle between the delaminating strip and the substrate throughout the entire test. The average peel force was determined from tests conducted on five separate samples. The 90° peel test was also repeated on samples that had undergone seed layer etching and/or annealing to determine the adhesion strength between the metal and the glass after these post-processing steps.

The grain structure of the copper films before and after annealing was characterized using a high-resolution X-ray diffractometer (D8 DISCOVER, Bruker, Billerica, MA, USA). The cross-sectional morphology of the copper films was observed using an FIB-SEM ultra-high-resolution dual-beam processing microscopy system (Scios2 HIVac, Thermo Fisher Scientific, Waltham, MA, USA).

## 3. Results and Discussion

### 3.1. Microscale Characterization and Mechanism Study of Glass Surfaces Activation

Among various surface modification techniques, plasma treatment is highly favored due to its efficiency, cleanliness, environmental friendliness, and ease of integration into existing production lines. Through bombardment by energetic particles, it effectively removes surface organic contaminants and introduces new functional groups, preparing the surface for enhanced bonding performance. Compared to nitrogen plasma, oxygen plasma treatment can activate the glass surface more significantly and improve bonding capability [9,33]. Therefore, oxygen plasma was selected for surface activation. The elemental states of the glass surface before and after treatment were analyzed by XPS, revealing the underlying mechanism of surface activation.

**Figure 2:**
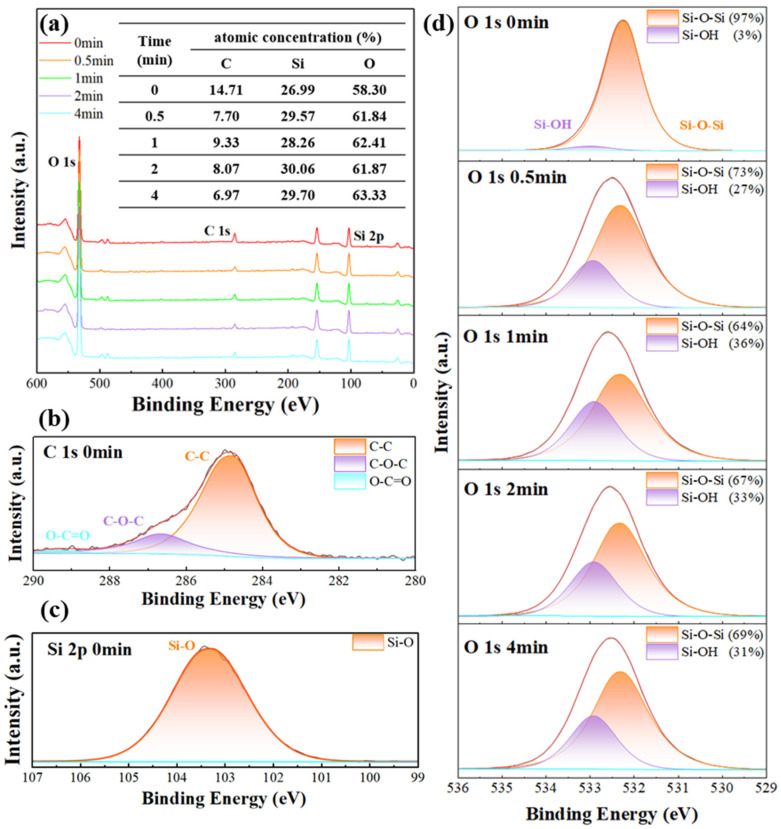
XPS spectra of the glass surface: (**a**) Survey spectra after different treatment durations, with the table showing the relative atomic concentrations of C, O, and Si; (**b**) C 1s and (**c**) Si 2p spectra of the untreated glass; (**d**) O 1s spectra after different treatment durations (showing the relative contribution of Si-O-Si and Si-OH).

Taking the 1 min plasma treatment as an example, the relative atomic concentration of carbon decreased from 14.71% before treatment to 9.33% after treatment, while those of silicon and oxygen increased from 58.30% and 26.99% to 62.41% and 29.57%, respectively (Figure 2a). This confirms the cleaning effect of plasma treatment and the introduction of more oxygen-containing functional groups. Note that the atomic percentages shown in the figure are normalized using atomic sensitivity factors, representing the elemental percentages of the respective species [34].

The C 1s spectrum was used for charge correction. For the silicon spectrum, the Si 2p_3/2_ and Si 2p_1/2_ peaks overlap in XPS, and Si-OH groups manifest only as a broadening of the Si 2p peak, making them indistinguishable from the Si-O-Si network [35]. Therefore, only the C and Si spectra of the untreated glass surface are presented here, without further analysis (Figure 2b,c).

For the O 1s spectrum, the O 1s peak primarily consists of oxygen from siloxane bonds (Si-O-Si, bridging oxygen), silanol bonds (Si-OH) and water. In the vacuum environment of XPS measurement, water readily desorbs from the sample surface. Therefore, the hydroxyl groups on the sample surface can be attributed to silanol groups within the inorganic polymer. The O 1s peak was fitted using two components: Si-O-Si (532.2 eV) and Si-OH (532.9 eV) (Figure 2d) [36]. Based on electrostatic considerations, the electronegativity of hydrogen is greater than that of silicon. The average electron count on the oxygen atom in Si-OH is lower than in Si-O-Si, reducing electron–electron repulsion and resulting in a higher electron binding energy. For the untreated glass, the signal intensity of Si-OH is very weak and almost negligible. As the oxygen plasma treatment duration increased, the relative content of Si-OH first rose significantly, reaching a maximum (36%) at 1 min, and then gradually decreased. The bombardment by energetic particles causes the breaking of Si-Si and Si-O-Si bonds. Hydroxyl groups bond with the exposed silicon atoms, and surface oxygen atoms also undergo hydrogenation, leading to surface hydroxylation of the glass and an increase in Si-OH content [37,38]. Concurrent with surface hydroxylation, the glass surface is continuously etched and damaged. This exposes new inert functional groups beneath the activated layer, forming a new surface layer and increasing surface roughness, which prevents further increase in Si-OH content [37].

**Figure 3:**
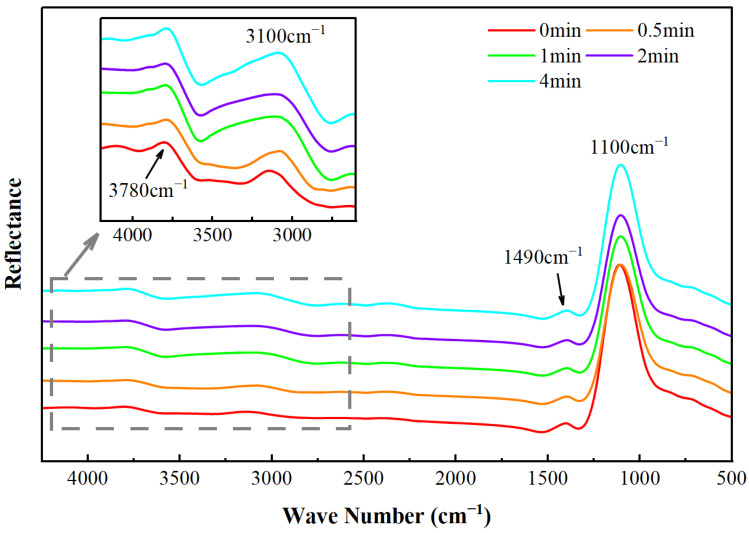
Reflection FTIR spectra under different treatment durations (the inset shows the magnified view of the 2600 cm^−1^–4200 cm^−1^ range).

In addition to XPS characterization, the reflection FTIR spectra also illustrate the changes in the chemical state of the glass surface (Figure 3). The intense signal at 1100 cm^−1^ corresponds to the intrinsic vibrational frequency of the Si-O-Si bonds in the glass surface region [39]. A smaller peak is observed at 1490 cm^−1^. Since the reflection spectra were measured in an ambient air environment, this spectral band corresponds to water adsorbed on the glass surface [40]. Due to the overwhelming intensity of the Si-O-Si signal, changes in other signals are not very pronounced. The spectral bands in the 2600–4200 cm^−1^ range were examined under magnification. The peak at approximately 3780 cm^−1^ corresponds to isolated free hydroxyl groups and terminal hydroxyl group of the H-bonded species [41]. The broad peak at 3100 cm^−1^ is attributed to the stretching vibrations of hydrogen-bonded hydroxyl groups and adsorbed water. With increasing treatment time, the peak at 3780 cm^−1^ gradually becomes sharper, while the peak at 3100 cm^−1^ progressively broadens and increases in intensity. This reflects the growing significance of hydroxyl groups, indicating that plasma treatment hydroxylates the glass surface, generating more silanol groups (Si-OH).

**Figure 4:**
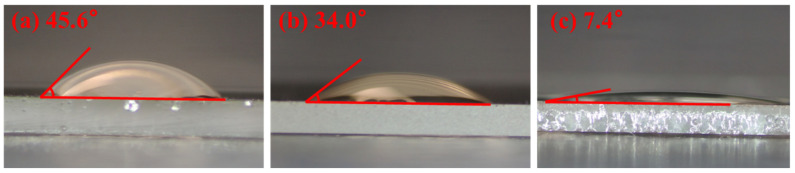
Surface water contact angle of the glass: (**a**) Untreated (45.6°); (**b**) After cleaning (34.0°); (**c**) After plasma treatment (7.4°).

At the macroscopic level, the water contact angle reflected the changes in surface energy induced by the modification treatment. The water contact angle of the cleaned glass decreased from 45.6° (uncleaned, Figure 4a) to 34.0° (Figure 4b), indicating a reduction of low-polarity organic residues and improved wettability. After 1 min of oxygen plasma treatment, the water contact angle sharply decreased to 7.4° (Figure 4c), indicating a dramatic increase in glass hydrophilicity. This is attributed to the further removal of organic matter and the generation of polar dangling bonds, which corresponds to the formation of additional hydroxyl groups as evidenced by XPS and FTIR characterization results [42]. Consequently, after cleaning and plasma treatment, the glass samples exhibited higher surface hydrophilicity, increased surface energy, and more polar functional groups, which facilitate interactions between the glass and metal.

**Figure 5:**
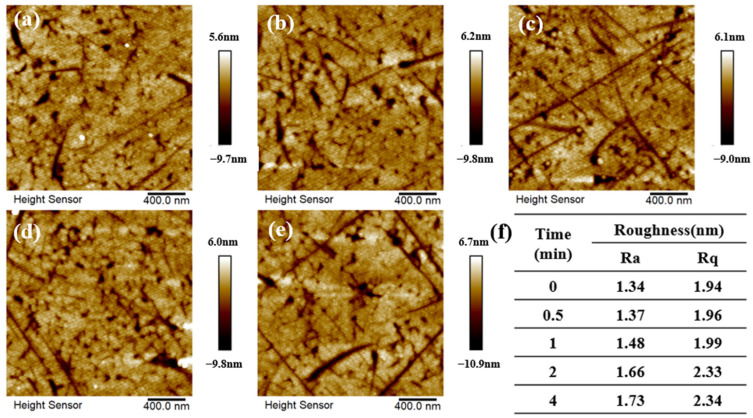
Surface morphology changes after different treatment durations: (**a**) 0 min, (**b**) 0.5 min, (**c**) 1 min, (**d**) 2 min, (**e**) 4 min, (**f**) Surface roughness data.

Furthermore, AFM was employed to characterize the evolution of surface roughness and morphology of the glass. As the treatment duration increased, the surface roughness of the glass continuously rose from an initial value of 1.34 nm (Ra) to 1.73 nm (Ra) of 4 min, reflecting the etching effect of the plasma treatment on the glass surface (Figure 5). However, the roughness remained at the nanometer level and did not increase dramatically.

In summary, the activation mechanism of plasma treatment on the glass surface can be elucidated through microscale characterization. While cleaning the glass surface, the bombardment of high-energy particles induces surface hydroxylation, leading to a significant increase in oxygen-containing functional groups (Si-OH), with a peak relative content of 36%. This may be beneficial to the interaction between glass and metal atoms, facilitating the formation of a bonding layer at the interface. Meanwhile, the increase in polar components can also enhance the electrostatic interaction between the metal and the glass substrate [26,43]. Additionally, greater surface roughness provides a larger contact area between the glass substrate and the metal, which is conducive to promoting mechanical interlocking between them [44,45]. Under the sample dimensions and experimental conditions employed in this study, a plasma treatment time of 1 min was selected as the optimal surface treatment condition for subsequent experiments, ensuring a sufficient number of polar functional groups on the surface while maintaining an appropriate level of roughness.

### 3.2. Correlation Between the Microscopic Bonding Mechanism and the Macroscopic Adhesion Strength

**Figure 6:**
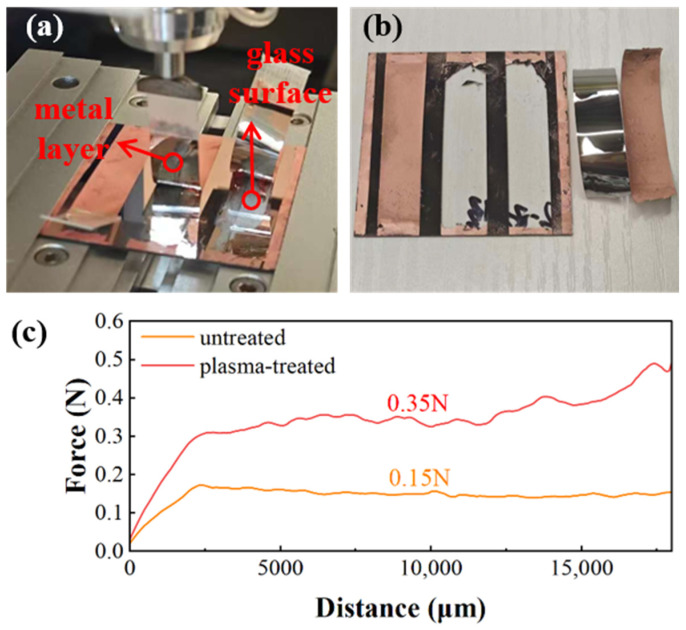
The 90° peel test: (**a**) Optical photograph during the test and (**b**) after the test, showing the substrate and metal strip; (**c**) test results. The adhesion strength value of the electroplated Cu is the average of multiple tests, with one representative curve shown.

Based on the surface hydroxylation effect revealed in Section 3.1, this section further investigates the interfacial bonding behavior between activated glass surface and Ti seed layer, and discusses its correlation with macroscopic adhesion strength. After electroplating, the adhesion between the thicker copper strips and the glass substrate was quantitatively tested (Figure 6a,b). The 90° peel test results showed that the average adhesion strength increased from 0.15 ± 0.02 N/cm for the untreated samples to 0.35 ± 0.03 N/cm for the treated samples (Figure 6c), achieving a 133% enhancement. After peeling, the glass side revealed a smooth, transparent glass surface, while the seed layer in contact with the glass exhibited a silver–gray color characteristic of Ti. Therefore, it can be inferred that the failure occurred at the glass–Ti seed layer interface.

**Figure 7:**
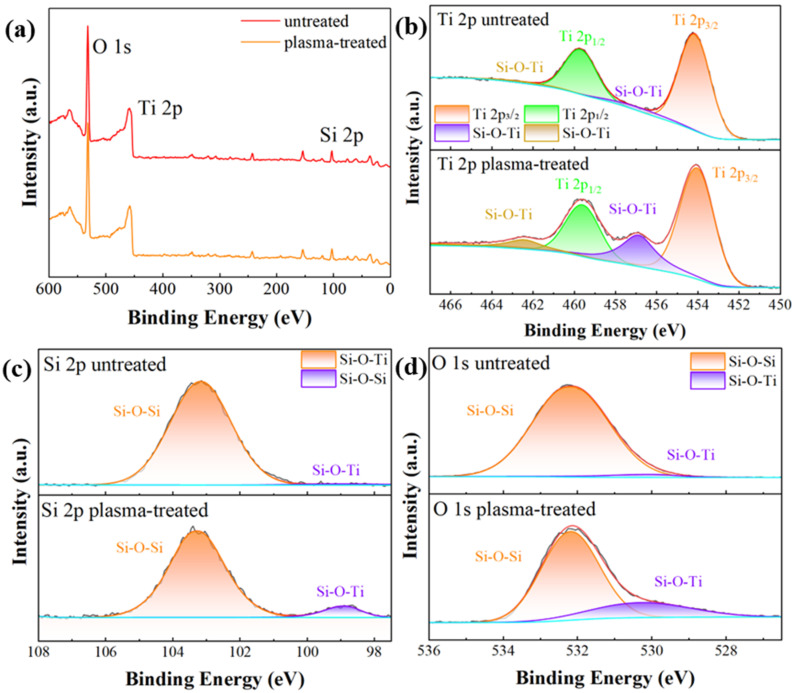
XPS spectra of the glass–Ti interface before and after plasma treatment: (**a**) survey spectra, (**b**) Ti 2p, (**c**) Si 2p and (**d**) O 1s spectra.

To further analyze the influence of plasma treatment on the evolution of bonding behavior at the glass–metal interface and to understand the mechanism behind the resulting improvement in interfacial adhesion strength, elemental information from the glass–Ti interface before and after plasma treatment was collected via XPS depth profiling. Figure 7a shows the survey spectra at the interface. Compared to the survey spectra before seed layer sputtering (Figure 2a), a strong characteristic Ti peak is evident around 460 eV. For the sample before treatment, the Ti fine spectrum was successfully fitted using a doublet at 454.1 eV (Ti 2p_3/2_) and 459.9 eV (Ti 2p_1/2_), corresponding to the chemical state of metallic titanium (Figure 7b) [46]. The Ti spectrum after treatment was distinctly different, showing a doublet at higher binding energies, which surpasses the features of typical metallic titanium and can be attributed to an oxidized state of Ti. A successful fit was achieved using a doublet at 457.5 eV and 462.5 eV, which is consistent with the characteristic peak of Si-O-Ti bonds (Figure 7b) [9]. No significant Si-O-Ti signal was observed in the spectra before treatment.

To further illustrate the impact of surface treatment on interfacial bonding, the Si 2p and O 1s fine spectra were also deconvoluted (Figure 7c,d). For the untreated sample, the fine spectra of Si and O at the interface showed curves similar to those before metal sputtering, with peaks at 103.2 eV and 532.3 eV corresponding to Si-O-Si. After treatment, distinct signals for the Si-O-Ti cross-link were also observed at 98.9 eV (Si 2p) and 530.1 eV (O 1s), which supports the formation of Si-O-Ti covalent bonds at the interface [47].

The results presented in the previous section collectively reveal the chemical state evolution at the interface following metal deposition. The plasma treatment successfully induced surface hydroxylation, providing an ideal precursor for interfacial reactions. The generated surface Si-OH groups likely undergo condensation reactions with the deposited titanium layer, forming Si-O-Ti covalent bonds. However, the specific reaction pathway remains to be further clarified by additional experimental investigation. The resulting chemical bonds create molecular bridges at the interface, transforming the originally van der Waals-dominated heterojunction into a bonding mode that incorporates chemical linkages. This transformation contributes, to some extent, to the enhanced adhesion between the entire metallization layer and the substrate after electroplating. Therefore, it can be inferred that the evolution of the interfacial chemical state observed through microscopic characterization techniques such as XPS and FTIR correlates with the improvement in interfacial adhesion measured in macroscopic mechanical tests. This correlation provides insight into the underlying mechanism behind the enhanced adhesion. The findings suggest that the increase in interfacial adhesion can be attributed, in part, to plasma-activated interfacial chemical bonding.

### 3.3. Influence of Annealing and Micro-Etching Processes on Adhesion

**Figure 8:**
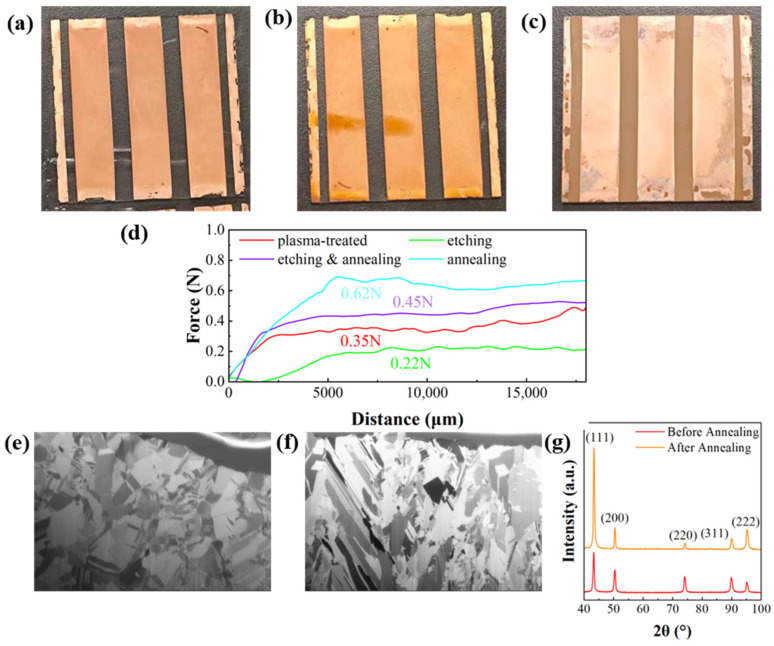
Photographs of (**a**) an etched sample, (**b**) an etched & annealed sample and (**c**) an annealed sample; (**d**) adhesion strength test curves of the electroplated Cu for samples subjected to etching, annealing, and etching & annealing; (**e**) FIB cross-sectional image of the copper film before annealing; (**f**) FIB cross-sectional image of the copper film after annealing; (**g**) XRD characterization results of the copper film.

In semiconductor packaging manufacturing, after electroplating copper lines for electrical interconnection, seed layer etching is typically performed to isolate individual copper line patterns and prevent short circuits [48]. Annealing is also commonly employed to improve the copper’s microstructure and relieve internal stresses [49]. To evaluate the impact of these process steps on interfacial adhesion, the electroplated samples underwent seed layer etching and/or annealing. Figure 8a–c show photographs of the samples after etching, etching & annealing, and annealing, respectively.

The interfacial adhesion strength was then measured via the 90° peel test (Figure 8d). The results show that after annealing alone, the average adhesion strength between the copper strips and the glass substrate increased to 0.62 ± 0.04 N/cm. After etching alone, the adhesion strength decreased to 0.22 ± 0.02 N/cm. However, subsequent annealing of the etched samples restored the adhesion strength to 0.45 ± 0.03 N/cm.

For the etched samples, during the peeling process, only the patterned copper strips separated from the glass substrate. The force overcome is primarily the interfacial adhesion strength. For the non-etched samples, during peeling, the copper strips in the electroplated areas detach, but the seed layer metal in the non-electroplated areas remains on the substrate (Figure 6b). Thus, the peel force needs to overcome not only the interfacial adhesion but also the cohesive force within the seed layer, causing the metal to tear between the electroplated and non-electroplated regions. This is the reason for the slight decrease in measured adhesion strength after seed layer etching.

The FIB cross-sectional images reveal the morphology of the orientation of crystal grain copper film before and after annealing (Figure 8e,f). Combined with the texture changes characterized by XRD (Figure 8g), the as-deposited Cu film exhibited five primary orientations: (111), (200), (220), (311), and (222). Before annealing, these five textures were present in comparable proportions, indicating that the copper film consisted of a uniform mixture of grains with these five orientations. After annealing, the proportion of the (111) texture increased significantly, becoming the dominant preferred orientation, while the other textures were present only in minor amounts. This demonstrates that annealing induced a texture transformation. The copper microstructure underwent recrystallization, where irregular small grains transformed into larger ones, resulting in increased grain size. It can be inferred that a mismatch in the coefficient of thermal expansion (CTE) between the glass and metal, along with intrinsic stresses introduced during thin-film deposition, generates residual stresses at the interface [12,50]. These stresses create shear or peeling forces, forming weak points in the bond. For the metallic copper, annealing activates atomic diffusion and dislocation motion, promoting recovery and recrystallization within the material. This process transforms its microstructure from one characterized by coarse columnar grains or high defect density into a uniform, fine-grained equiaxed structure. These new grains grow until they completely replace the old, distorted grain structure. Consequently, the internal stress is relaxed, leading to a more uniform stress distribution near the interface and thereby mitigating the mechanical driving force for interfacial failure. Thus, after annealing, the residual stress in the coating is alleviated, resulting in enhanced adhesion at the glass–metal interface.

In summary, in addition to the interlayer fabrication or surface pretreatment processes examined in previous studies, post-electroplating processes such as seed layer etching and annealing also influence the adhesion strength of the glass–metal interface. On one hand, etching removes the seed layer, leading to the loss of intermolecular forces between metals and consequently reducing adhesion strength. On the other hand, annealing alters the grain orientation of the metal and relieves stress within the plated layer, thereby enhancing adhesion strength.

## 4. Conclusions

This study uses atomic-scale characterization methods to investigate the effects of surface modification on the chemical state and elemental composition of glass surfaces, and analyzes the corresponding modification mechanisms. The evolution of interfacial chemical states at the glass–metal interface after seed layer deposition is further examined, and the influences of post-processing steps on interfacial adhesion are discussed. The main findings and conclusions are as follows:

1. Oxygen plasma treatment induces surface hydroxylation on glass, and the relative content of Si-OH groups reaches a maximum of 36% under the experimental conditions. This surface modification process is characterized and verified by XPS analysis at the atomic scale.
2. FTIR characterization reflects the evolution of surface chemical states on glass substrates after plasma treatment. The results are consistent with XPS findings, both confirming the increase in hydroxyl group content after surface treatment.
3. Si-O-Ti covalent bonds are formed at the glass–titanium interface after Ti deposition on the plasma-activated glass surface. The formation of this interfacial bonding configuration is correlated with the enhancement of macroscopic adhesion strength.
4. Seed layer etching and annealing lead to a decrease and an increase in glass–metal adhesion, respectively. Combined with microscopic microstructure characterization and macroscopic adhesion tests, the possible mechanisms behind these variations are discussed. Under the optimized process conditions, the maximum adhesion strength reaches 4.1 times that of the initial untreated sample.

In summary, this work is a fundamental mechanistic study focusing on glass surface activation, interfacial chemical bonding and post-process adhesion regulation at the microscale.

It should be noted that this work is currently limited to microscopic mechanism analysis of glass surface modification and basic room-temperature adhesion characterization, and does not involve packaging-level reliability evaluations including thermal cycling, high-temperature humidity exposure and long-term aging tests. For subsequent research in this field, systematic reliability tests relevant to packaging applications are recommended to investigate the long-term stability and interfacial failure mechanisms of glass–metal interfaces under actual service conditions.

The findings of this work clarify the intrinsic correlation between microscopic interfacial evolution and macroscopic adhesion performance and can provide mechanistic reference for further research on glass–metal interfacial modification.

## Data Availability

The data presented in this study are available on request from the corresponding author.
